# Stop, neighbor! KLU–PREs positional signaling restricts female germline fate in Arabidopsis

**DOI:** 10.1093/plcell/koag038

**Published:** 2026-02-20

**Authors:** Jiajun Wang

**Affiliations:** The Plant Cell, American Society of Plant Biologists; School of Life Sciences, Xiamen Key Laboratory of Plant Genetics, Xiamen University, Xiamen 361102, China

The ability to specify a single megaspore mother cell (MMC) within the ovule primordium, while preventing neighboring somatic cells from acquiring germline fate, is essential for successful sexual reproduction in plants. This tightly regulated process establishes the female germline and sets the developmental trajectory for subsequent female gametophyte formation in flowering plants (Huang et al. 2024). While complex signaling networks ensure the singular MMC fate, how surrounding somatic nucellar cells maintain their identity remains unclear. In new work, **Hanyang Cai and colleagues** (Cai et al. 2026) elucidate a crucial positional signaling module involving KLU and the PRE family that is orchestrated by the brassinosteroid-responsive transcription factor BZR1 enforces this developmental fidelity (Fig. 1).

**Figure 1:**
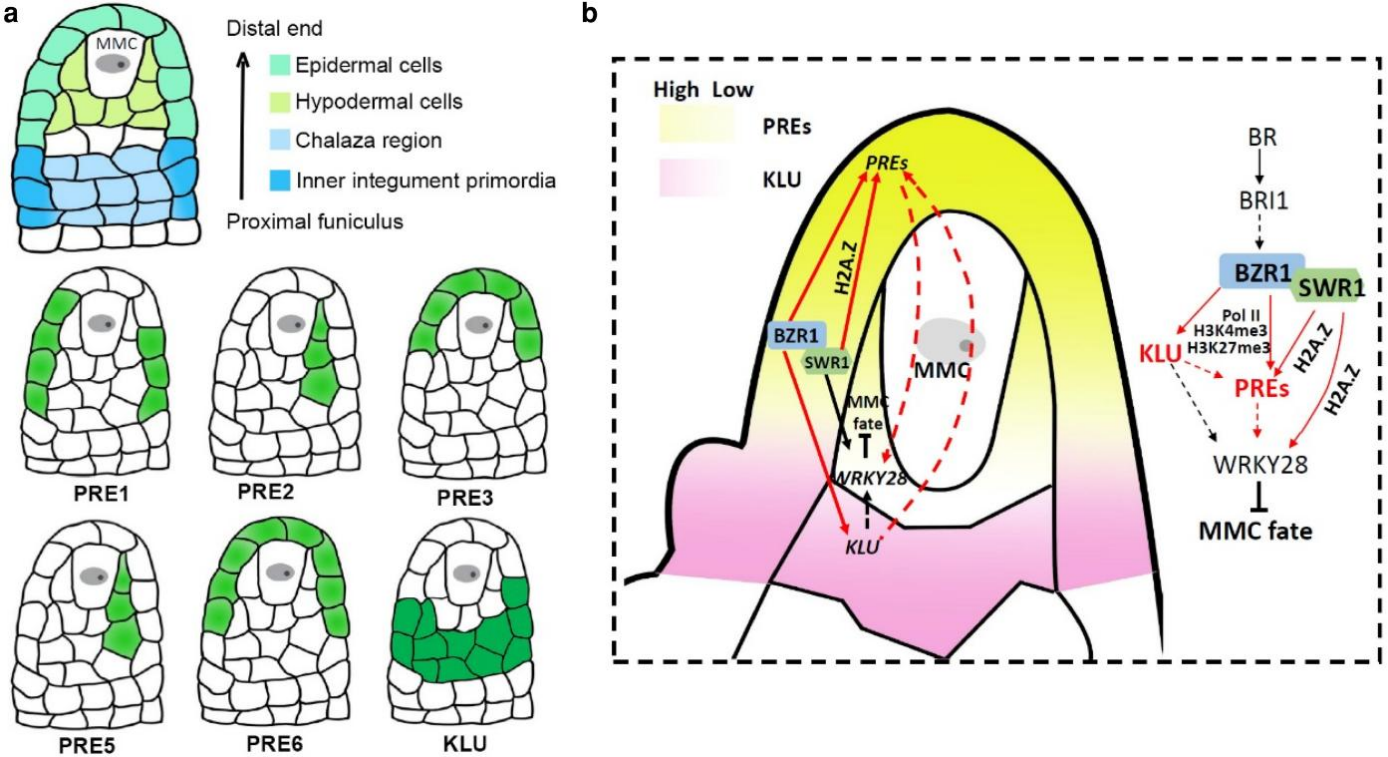
Schematic diagram of the spatially specific expression of PREs and KLU in controlling MMC specification within the ovule primordium. **(a)** Schematic representation of PRE1/2/3/5/6 and KLU protein expression patterns and subcellular localization during early ovule development. **(b)** Model depicting the upstream regulation of PREs and KLU by brassinosteroid (BR) signaling and chromatin remodeling. BZR1 promotes *PRE* gene expression in epidermal or hypodermal nucellar cells through the SWR1 complex and KLU, while concurrently activating *KLU* expression in the inner integument primordia and chalaza region. This spatial regulation restricts PRE protein spread into these proximal domains, thereby maintaining somatic cell identity and ensuring the specification of a single MMC. Modified and reprinted from Figures 1 and 7 of Cai et al. (2026).

The PACLOBUTRAZOL-RESISTANCE (PRE) gene family encodes 6 atypical, likely non–DNA-binding basic helix–loop–helix (bHLH) proteins (PRE1–PRE6) that integrate multiple signaling pathways to regulate cell elongation as well as floral organ growth and development (Shin et al. 2019). Cai et al. found that generating a *PRE* quintuple mutant (*pre-quin*) via CRISPR-mediated knockout of *PRE3* in a *pre1 pre2 pre5 pre6* artificial microRNA knockdown background (*pre-amiR*) resulted in sterility, with approximately 35% of ovules exhibiting a multiple MMC-like specification phenotype. In contrast to *KLU*, a cytochrome P450 enzyme that is primarily expressed in the inner integument primordia and the chalaza region of the proximal funiculus, *PRE1/2/3/5/6* are preferentially expressed in the epidermis (*PRE1/3/6*) and hypodermis (*PRE2/5*) of distal nucellar cells surrounding the MMC. This complementary expression pattern is essential for restricting MMC fate. In this work, ectopic expression of *PRE1/2/3/5/6* in the inner integument primordia and chalaza region, but not in other regions, significantly increased the proportion of hypodermal cells that differentiated into MMC-like cells. Conversely, ectopic expression of *KLU* in the distal epidermis and hypodermal MMC-surrounding cells also induced the formation of extra MMC-like cells. These results indicate that the spatially restricted expression of *PREs* in distal epidermal and hypodermal cells, together with KLU expression in the inner integument primordia and chalaza region, is crucial for limiting distal nucellar cell fate to a single MMC.

Interestingly, PRE proteins have previously been found to spread to neighboring cells during early ovule development (Lu et al. 2018). The authors therefore investigated why PRE proteins normally fail to extend into the inner integument primordia and chalaza region. In *klu* mutants, the spatiotemporal expression patterns of *PREs* were unaffected; however, PRE proteins spread ectopically from distal epidermal or hypodermal MMC-surrounding cells into the inner integument primordia and chalaza region. KLU directly interacts with PREs and likely inhibits PRE protein spread into these proximal domains by acting on the conserved M8 motif of PREs. Artificially confining PREs to their native domain rescued the multiple-MMC phenotype of *klu* mutants, confirming the functional importance of this spatial restriction. Consistently, knocking down *PRE1/2/5/6* in the *klu* mutant background significantly increased the proportion of ovules producing multiple MMC-like cells and resulted in sterility.

To investigate the upstream mechanisms controlling the distinct positional expression of *PREs* and *KLU* in the ovule primordium, the authors focused on BZR1-family transcription factors. Brassinosteroids have previously been implicated in female germline specification, and the *bzr1 bes1 beh1 beh3 beh4* quintuple mutant (*qui-1*) displays more than 70% of ovules containing multiple MMCs (Cai et al. 2022). The authors showed that BZR1 binds directly to E-box motifs in the promoters of *PRE1/2/3/5/6* and *KLU*, thereby activating their transcription. Introducing a *klu* loss-of-function mutation into 5 different combinations of BZR1-family quadruple mutants significantly increased the frequency of ovules containing enlarged MMC-like cells compared with either the respective quadruple mutants or the *klu* single mutant.

In addition, BZR1 was found to physically interact with SWC6, a subunit of the SWR1 chromatin remodeling complex, and SWC6 and KLU genetically interact to suppress supernumerary MMC specification. BZR1 binding to the *PRE1* promoter was shown to be partially dependent on SWC6 and KLU. Similar to the BZR1-family quintuple mutant (*qui-1*), the *swc6 klu* double mutant exhibited markedly reduced RNA polymerase II occupancy, H3K4me3 levels, and H2A.Z deposition near the transcription start sites of *PRE1/2/3/5/6*, accompanied by a pronounced increase in H3K27me3. These chromatin changes correlated with a strong reduction in *PRE1/2/3/5/6* transcription in the *swc6 klu* background, indicating that BZR1-dependent *PRE* activation partially relies on SWC6- and KLU-mediated chromatin regulation.

In summary, by integrating detailed cytological observations with molecular and genetic analyses, the authors uncover a hierarchical regulatory network in which the precise spatial expression of PREs and KLU in the ovule primordium suppresses germline potential in surrounding somatic nucellar cells. BZR1 executes this control through 2 parallel pathways: it promotes PRE transcription in distal epidermal and hypodermal cells in a manner partially dependent on SWC6 and KLU, while simultaneously activating *KLU* expression in the inner integument primordia and chalaza region to prevent PRE protein spread into this domain. This coordinated spatial regulation ensures the robust restriction of MMC fate to a single cell during ovule development. Collectively, this work elegantly integrates hormone signaling, transcriptional control, chromatin dynamics, and protein mobility to explain a fundamental patterning event in plant reproduction.

## Recent related articles in *The Plant Cell*

- Jiang et al. (2025) revealed that a precise balance between DNA methylation and demethylation, rather than absolute methylation levels, maintains the specification of a single megaspore mother cell (MMC) by ensuring differential mCHH hypomethylation in the MMC precursor compared to its somatic neighbors.
- Wang et al. (2024) demonstrated that brassinosteroid signaling spatially restricts the expression of the AGAMOUS-activated gene *ZINC FINGER PROTEIN 11* in the chalaza and nucellus via the transcription factor BZR1, thereby orchestrating integument development and controlling ovule number to ensure proper female reproductive structure formation.
- Shi et al. (2024) identified that the adaptor protein ECAP forms a transcriptional activator complex with the corepressor LEUNIG and the transcription factor BEH3 that epigenetically regulates *SPOROCYTELESS* expression to control archesporial cell specification and microsporocyte generation during early anther development.

## Dive Curated Terms

The following phenotypic, genotypic, and functional terms are of significance to the work described in this paper:

PRE1 Gramene: AT5G39860
PRE1 Araport: AT5G39860

MMC AmiGo: PO:0000431

SWC6 Gramene: AT5G37055
SWC6 Araport: AT5G37055

BZR1 Gramene: AT1G75080
BZR1 Araport: AT1G75080

BEH3 Gramene: AT4G18890
BEH3 Araport: AT4G18890
